# A cross-lagged analysis of the relationship between marital quality and depression among the older adults: gender effects of socioeconomic status

**DOI:** 10.3389/fpsyg.2025.1389801

**Published:** 2025-01-15

**Authors:** Bi-Ji Fang, Kai-Seng Leong, Hong-Xiu Tan

**Affiliations:** ^1^School of Education Science, Shaoguan University, Shaoguan, Guangdong, China; ^2^Faculty of Health and Wellness, City University of Macau, Taipa, Macao SAR, China

**Keywords:** the older adults, marital quality, depression, socioeconomic status, gender effect

## Abstract

**Objective:**

This study aimed to explore the mechanism and gender effect of socioeconomic status on the relationship between marital quality and depression among the older adults, with the intention of providing a practical foundation for enhancing the quality of life of the older adults.

**Methods:**

The data sourced from the third (conducted in 2015, denoted as the first survey) and fourth (carried out in 2018, regarded as the second survey) installments of the China Health and Retirement Longitudinal Survey (CHARLS) were meticulously analyzed through the utilization of cross-lagged analytical techniques and moderating effect examination methodologies.

**Results:**

Among the older adults, there exists a reciprocal causal relationship between marital quality and the level of depression. Specifically, the marital quality as measured in the first survey was found to significantly prognosticate the depression level in the second survey (*β* = 0.05, *p* < 0.05). Conversely, the depression level in the first survey was also demonstrated to significantly forecast the marital quality in the second survey (*β* = 0.15, *p* < 0.01). Regarding the moderating role of socioeconomic status, it was observed that among the older adult women, socioeconomic status exerted a moderating influence on the impact of depression on marital quality (*β* = 0.05, *p* < 0.05), whereas among the older adult men, their socioeconomic status failed to display a significant moderating effect (*β* = 0.02, *p* > 0.05). In a contrasting manner, for the older adult men, the socioeconomic status played a moderating role in the effect of marital quality on depression (*β* = −0.05, *p* < 0.01), while for the older adult women, it did not exhibit a moderating effect (*β* = −0.01, *p* > 0.05).

**Conclusion:**

There exists a reciprocal causal nexus between the marital quality and depression among the older adults. To enhance the life quality of the older adults, it behooves communities and families to proactively focus more on the older adults with subpar marital quality or afflicted by depression, and to disrupt the cyclic interplay between these two variables.

## 1 Introduction

In recent years, against the backdrop of the rapid economic advancement and the enhanced medical care, the issue of global population aging has been thrust into sharper relief, emerging as a major social conundrum that merits in-depth exploration and vigilant attention (Lopreite and Zhu, 2020; Ling et al., 2023). China, as a densely populated nation, is grappling with a particularly acute aging problem. As per the “National Aging Development Bulletin 2022” issued by the Ministry of Civil Affairs of the People’s Republic of China, by the close of 2022, the proportion of the population aged 60 and above in China had reached 19.8% (Ministry of Civil Affairs of the People’s Republic of China, 2022). With the march of age and the attenuation of physical faculties, the physical well-being of the elderly is generally compromised, frequently accompanied by chronic ailments (Krick et al., 2022). The waning of cognitive capabilities precipitates a commensurate reduction in social engagement and a concomitant spike in solitary hours for the older adults (Jiang and Liu, 2023). These elements exert a pronounced detrimental influence on the psychological health of the elderly, predisposing them to adverse emotions such as loneliness and depression (Tseng et al., 2019; Dinius et al., 2023; Wang D. et al., 2022; Hu et al., 2022).

### 1.1 The prevalence and detrimental impacts of depression among the older adults

Studies have demonstrated that depression has emerged as one of the prevalent mental disorders afflicting the older adults in China (Jia et al., 2020; He et al., 2022). Surveys suggest that the rate of detecting depressive symptoms among the Chinese older adults hovers around 20% (Wang X. et al., 2022; Wang et al., 2022). Notably, the older adults who reside alone and those grappling with chronic ailments exhibit more conspicuous depressive propensities (Wang et al., 2019; Chai et al., 2024). Analogous circumstances are also observable in other Southeast Asian nations. Research conducted in Malaysia reveals that the prevalence of depression among the Malaysian older adults is approximately 15% (Mulud and Mohamad, 2023). In particular, the older adults in rural regions, where medical resources are relatively scant and cultural and recreational pursuits are limited, are more susceptible to succumbing to depressive moods when confronted with the vicissitudes of life (Hamid et al., 2021). A study focused on the Thai older adults ascertained that roughly 18% of them experience depression to varying extents (Tangthong and Manomaipiboon, 2023). Simultaneously, under the influence of traditional Confucian values, a proportion of the older adults in Southeast Asian countries harbors biases against mental illnesses and demonstrates reluctance to proactively seek assistance. This has led to a multitude of depression cases going undetected and untreated in a timely fashion. Depression among the older adults not only impinges on their personal quality of life but also increases the burdens on both families and society at large.

### 1.2 The impact of marital quality among the older adults on depression

Research has indicated that marriage serves as an efficacious remedy for combating depression (Zhai et al., 2024). In accordance with the mechanism underlying the protective role of marriage, the daily care, financial support, accident prevention, social relationship upkeep, and spiritual solace provided by spouses exert a favorable safeguarding influence on the physical and mental well-being of the older adults (Kim et al., 2020; Zhao et al., 2023). However, the premise for this mechanism to take effect is that the couple possesses good marital quality. Research has indicated that couples with higher marital satisfaction are inclined to possess a relatively lower incidence of depression (Zhai et al., 2024). On the contrary, the incidence of depression among couples with poor marital quality may double (Robles et al., 2013).

A study conducted in Singapore has revealed that among the older adults whose marital quality ranks above the medium level, the percentage of those afflicted with depression is comparatively low; in contrast, within the older adults cohort where marital discord prevails, the prevalence of depression is likely to double (Tiong et al., 2013). Likewise, in other Southeast Asian nations, attributed to the rich family cultural milieu, the elderly with a more favorable marital condition tend to acquire more respect and solicitude within their families, consequently presenting a lower risk of depression (Liu et al., 2020). Nevertheless, for those elderly with unstable and troubled marriages, due to the dearth of a robust family support system, the incidence of depression exhibits a significant upsurge (Inaba et al., 2005).

Overall, the quality of marriage is particularly important for maintaining the physical and mental health of the older adults and improving their quality of life. Since most of the existing researches on the relationship between marital quality and depression are cross-sectional, it is difficult to reveal the causal relationship between these two variables, namely marital quality and depression (Bulloch et al., 2009; Lin et al., 2017; Dong et al., 2022). Simultaneously, owing to the sampling challenges, research centering on the relationship between marital quality and depression among the older adults has nearly turned into a neglected area.

To this end, this study will adhere to the concept of diachronic system design and employ a cross-lagged design to investigate the direction of the interaction between marital quality and depression among the elderly, and disclose the implied quasi-causal relationship therein. Previous studies have demonstrated that both marital quality and depression as variables are significantly affected by individuals’ socioeconomic status (Okhakhume et al., 2016; Du et al., 2021). Nevertheless, scant research has been conducted in academia regarding how socioeconomic status affects the relationship between marital quality and depression level among the older adults. Considering that Chinese older adults are deeply influenced by the traditional gender culture of “men are breadwinners while women are homemakers,” socioeconomic status holds different implications for older adults of different genders. Hence, it is hypothesized that there exists a gender effect in the impact of socioeconomic status between marital quality and depression level among the older adults. The present study aims to empirically investigate the aforementioned issues, hoping that the research findings will offer insights for enhancing the quality of life of the older adults and contribute to the attainment of a healthy aging society.

## 2 Data and methods

### 2.1 Data

The data utilized in this study were sourced from the third (conducted in 2015, the first wave of the survey) and fourth (conducted in 2018, the second wave of the survey) phases of the China Health and Retirement Longitudinal Survey (CHARLS), which were publicly available in October 2017 and September 2020, respectively. The CHARLS project, implemented by the National Development Research Institute at Peking University, employed scientific sampling techniques to gather comprehensive information on individuals aged 45 and above across the nation, encompassing details such as basic demographics, family circumstances, health conditions, physical examination outcomes, medical service utilization and insurance coverage, employment and retirement status, pension arrangements, income levels, consumption patterns, asset holdings, and community characteristics. For the present analysis, data were specifically selected from the responses of the same participants in both the 2015 and 2018 surveys. The inclusion criteria stipulated that all participants must have been 60 years of age or older in 2015 and possess valid data for both survey years. Participants with missing values pertaining to the variables under investigation in this study were excluded, yielding a final sample of 4,612 valid participants. Of these, 2,592 (56.20%) were male and 2020 (43.80%) were female. In the 2015 survey, the age range of the participants spanned from 60 to 92 years, with a mean age of 66.19 ± 5.17 years. Regarding educational attainment, 1,097 (23.79%) were illiterate, 1,090 (23.63%) were semi-illiterate, 1,217 (26.39%) had completed primary education, 995 (21.57%) had secondary education, and 213 (4.62%) had attained higher education.

### 2.2 Measurement of socioeconomic status

Socioeconomic status represents a comprehensive construct, conventionally employing education, income, and occupation as its proxy indicators (Galobardes et al., 2006). Since the majority of older adults have retired from the workplace, it is inappropriate to utilize the two indicators of occupation and income. The educational attainment level can signify an individual’s capacity to acquire resources and fulfill their personal needs. Hence, the educational level emerges as the most suitable indicator for gauging the socioeconomic status of the elderly. Consequently, the socioeconomic status of the older adults in this study was stratified into five levels: Level 1 was assigned to illiteracy, Level 2 to semi-illiteracy, Level 3 to primary education, Level 4 to secondary education, and Level 5 to higher education.

### 2.3 Measurement of marital quality

In the CHARLS project, a question was posed: “Are you satisfied with your marriage?” The participants were then asked to rate their level of satisfaction on a scale including “extremely satisfied,” “very satisfied,” “somewhat satisfied,” “not very satisfied,” and “not at all satisfied,” which were correspondingly assigned scores of 1, 2, 3, 4, and 5. It should be noted that, contrary to what might be expected, in this study, a higher score indicates a lower level of marital quality among the older adults.

### 2.4 Measurement of depression

In the CHARLS project, depression was gauged using the simplified version of the CES-D (Center for Epidemiologic Studies Depression Scale), which represents the most prevalently utilized depression scale in household surveys. The simplified CES-D comprises 10 items, each pertaining to a distinct scenario. The participants were queried by the researchers regarding the frequency of occurrence of these 10 scenarios within a week, with the responses categorized into four levels: rarely (less than a day), occasionally (1–2 days), frequently (3–4 days), and most of the time (5–7 days), and assigned scores of 0, 1, 2, and 3, respectively. The cumulative score was used to denote the depression level of the participants, spanning a range from 0 to 30 points. Notably, a higher score obtained by the participants corresponded to a greater degree of depression. A plethora of studies have indicated that the simplified version of the CES-D has manifested good reliability and validity upon application to the Chinese older adults (Zhang and Li, 2012; Ren et al., 2014; Guo et al., 2016). Hence, it is deemed as an effective tool for conducting surveys on the depression status of the elderly in China.

### 2.5 Statistical methods

In this study, SPSS 23.0 (IBM Corp, 2015) was employed to conduct descriptive statistics on the selected sample data. The principal indicators comprised the mean, standard deviation, and correlation coefficient. Subsequently, M-plus 8.3 was utilized to construct a cross-lagged model for the purpose of examining the longitudinal causal relationship between marital quality and depression level among the older adults. Eventually, the moderating effect of socioeconomic status on the relationship between marital quality and depression level among the older adults was analyzed through the Process V 3.4 plugin.

## 3 Results

### 3.1 Preliminary analysis on the data measured in 2015 and 2018

Utilizing the data collected in 2015 and 2018, the descriptive statistics as well as the Pearson product–moment correlation coefficients between marital quality and depression level among the older adults were computed. The results are presented in Table 1.

**Table 1:** Descriptive statistics and the Pearson product–moment correlation coefficients between marital quality and depression level among the participants (*N* = 4,612).

Variables	*M* ± *SD*	Marital quality (T1)	Marital quality (T2)	Depression (T1)
Marital quality (T1)	2.53 ± 0.78	1		
Marital quality (T2)	2.57 ± 0.80	0.40^**^	1	
Depression (T1)	17.80 ± 6.22	0.25^**^	0.24^**^	1
Depression (T2)	18.27 ± 6.49	0.18^**^	0.28^**^	0.55^**^

T1 represents the data collected in 2015; T2 represents the data collected in 2018. *P < 0.05, **P < 0.01 (two-tailed). The same applies hereinafter.

The results presented in Table 1 indicate that a high correlation exists between marital quality and depression among the older adults for the data measured in different years, with the correlation coefficient values being 0.40 and 0.55, respectively. It is also shown that the correlations between marital quality and depression, both within the same year and across different years, are statistically significant, with the lowest correlation coefficient being 0.18.

### 3.2 Cross-lagged analysis of marital quality and depression level among the older adults

**Figure 1:**
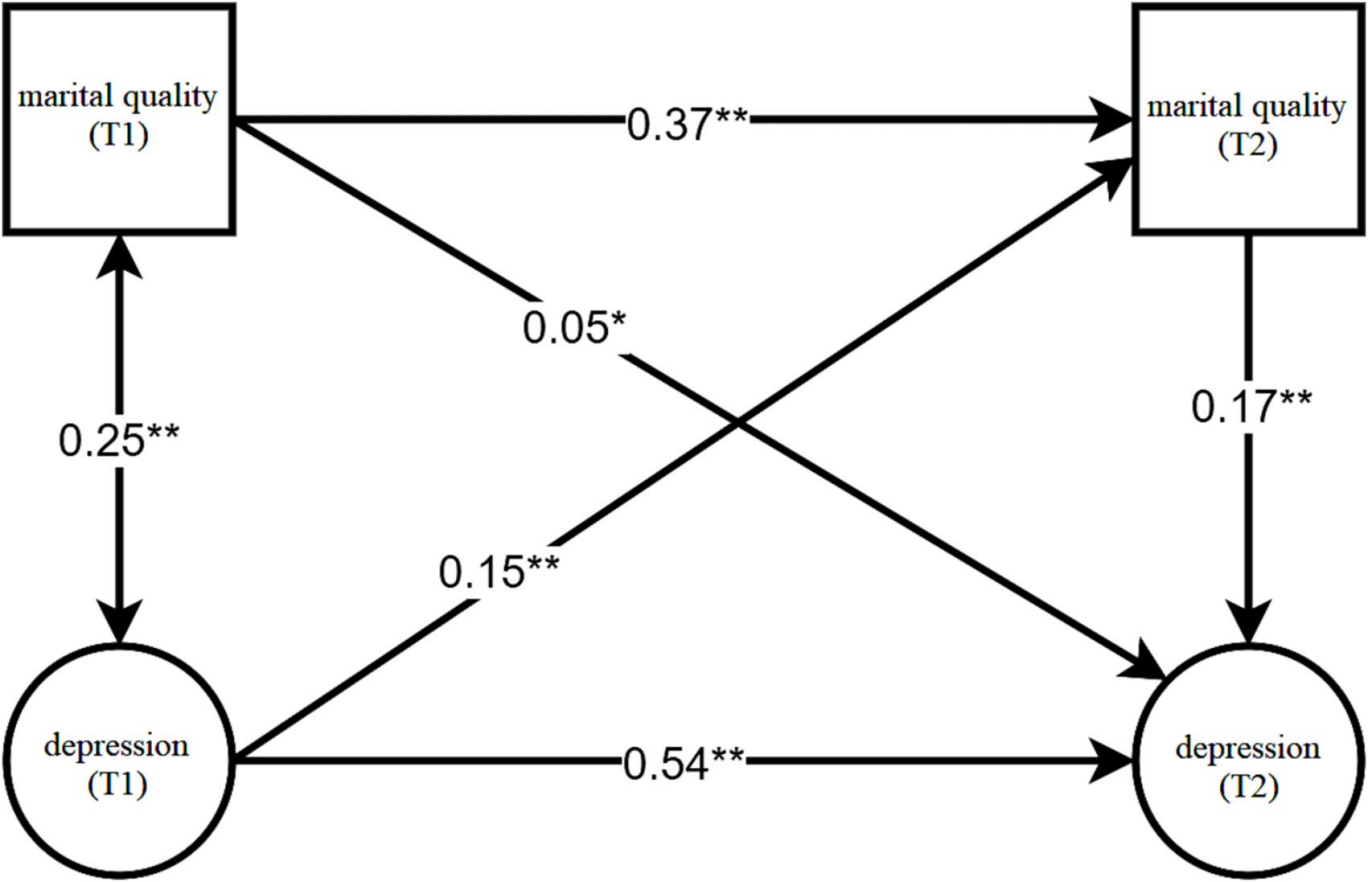
Cross-lagged analysis of marital quality and depression level among the older adults.

With gender, age, and education set as the control variables, a cross-lagged regression model was constructed using M-plus 8.3 to analyze the relationship between marital quality and depression level among the older adults. The model demonstrated good fit across various indicators (*χ*^2^/*df* = 17.68, *p* < 0.001, *CFI* = 0.85, *TLI* = 0.83, *RMSEA* = 0.06, *95%CI* = [0.059, 0.062], *SRMR = 0.05*). The path coefficients of the three covariates on marital quality (T2) were as follows: *β*_gender_ = 0.075 (*p* < 0.01), *β*_education_ = 0.017 (*p* > 0.05), *β*_age_ = 0.003 (*p* > 0.05). Meanwhile, the path coefficients of the three covariates on depression level (T2) were *β*_gender_ = 0.037 (*p* < 0.05), *β*_education_ = −0.087 (*p* < 0.01), *β*_age_ = 0.002 (*p* > 0.05). The path coefficients between marital quality and depression level among the older adults are depicted in Figure 1.

As depicted in Figure 1, within the autoregressive pathways, the marital quality measured in 2015 was found to positively predict that of the older adults measured in 2018. Similarly, the depression level measured in 2015 could positively predict the depression level of the older adults in 2018. Notably, the autoregressive coefficient of marital quality was lower than that of the depression level. In the cross-lagged regression paths, the marital quality measured in 2015 was demonstrated to positively predict the depression level of the older adults measured in 2018. In other words, a poorer marital quality in 2015 corresponded to a higher depression level in 2018 among the older adults, albeit with a relatively small path coefficient of 0.05. Conversely, the depression level measured in 2015 was shown to positively predict the marital quality of the older adults measured in 2018. That is, a higher depression level in 2015 was associated with a poorer marital quality in 2018, with a path coefficient of 0.15.

### 3.3 Analysis of the moderating effect of socioeconomic status on the relationship between depression level and marital quality among the older adults

It was revealed in the cross-lagged analysis that there exists a reciprocal causal relationship between marital quality and depression level among the elderly. Hence, we initially examined the role of socioeconomic status within the context of the “depression level → marital quality” relationship among the older adults. Previous research has indicated that an individual’s economic status can moderate their psychological resilience in the face of stressors. Specifically, those with a lower economic status tend to exhibit weaker psychological resilience and are less capable of effectively mitigating the negative consequences induced by stress events, thereby being more susceptible to depression (Peng et al., 2021). Consequently, we hypothesized that socioeconomic status serves as a moderator in the influence of depression on marital quality among the older adults.

The correlation analysis indicated that a moderate correlation exists between marital quality and depression level. Subsequently, the measurement scores of depression level and marital quality from the two rounds were averaged separately. With depression level serving as the independent variable, marital quality as the dependent variable, and socioeconomic status as the moderating variable, the analysis was conducted separately for each gender. The results are presented in Table 2.

**Table 2:** Moderating effect of socioeconomic status on the relationship between depression level and marital quality among the older adults (*N* = 4,612).

Gender	Variables	*β*	*S.E*	*t*	*P*	95% *CI*
Male	Depression	0.24	0.062	3.872	0.000	(0.118, 0.359)
	Socioeconomic status	0.02	0.017	0.874	0.382	(−0.019, 0.049)
	Socioeconomic status × depression	0.02	0.020	1.116	0.264	(−0.017, 0.063)
Female	Depression	0.34	0.053	6.385	0.000	(0.235, 0.444)
	Socioeconomic status	0.01	0.023	0.513	0.608	(−0.033, 0.057)
	Socioeconomic status × depression	0.05	0.023	2.022	0.043	(0.002, 0.092)

**Figure 2:**
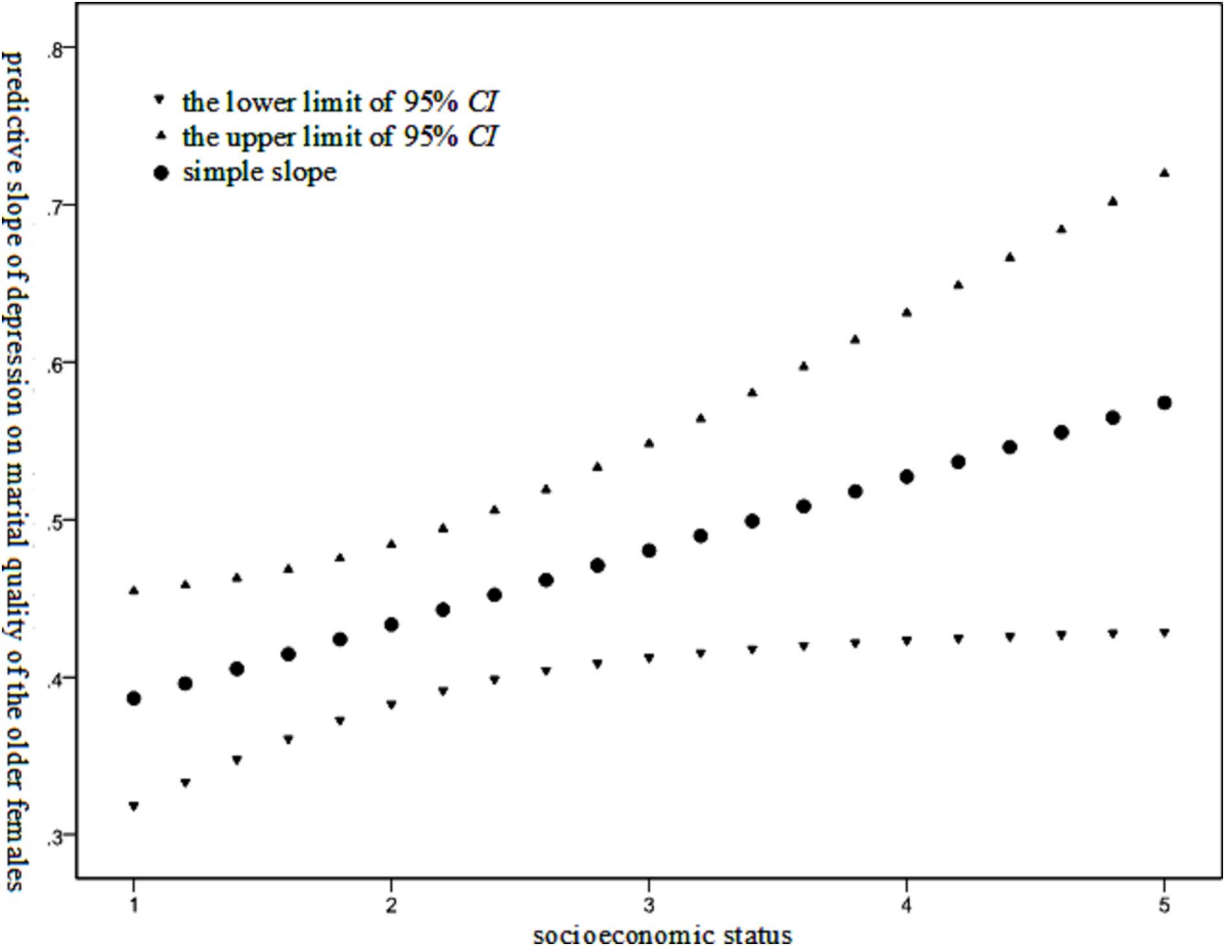
Moderating effect of socioeconomic status on the relationship between depression level and marital quality among the older females.

As indicated in Table 2, within the male group, only the variable of depression exerted a statistically significant effect on marital quality. In contrast, within the female group, both depression and the interaction between depression and socioeconomic status had an impact on marital quality, thereby suggesting that socioeconomic status plays a moderating role in the relationship between depression and marital quality among the older females. The Johnson-Neyman technique was employed to test the moderating effect of female socioeconomic status. The results are presented in Figure 2.

As illustrated in Figure 2, the negative effect of depression level on the marital quality of the older females was found to gradually increase with the improvement of their socioeconomic status.

### 3.4 Analysis of the moderating effect of socioeconomic status on the relationship between marital quality and depression level among the older adults

Generally speaking, individuals with higher socioeconomic status possess more health resources and social support, which implies that they are more capable of inhibiting the negative effects of physical and mental problems. Consequently, we hypothesized that socioeconomic status also serves as a moderator in the effect of marital quality on depression level among the older adults. With marital quality regarded as the independent variable and depression level as the dependent variable, the moderating effect of socioeconomic status on the relationship between marital quality and depression level among the older adults was analyzed separately according to gender. The results are presented in Table 3.

**Table 3:** Moderating effect of socioeconomic status on the relationship between marital quality and depression level among the older adults (*N* = 4,612).

Gender	Variables	*β*	*S.E*	*t*	*P*	95%*CI*
Male	Marital quality	0.34	0.048	7.145	0.000	(0.249, 0.437)
	Socioeconomic status	0.01	0.041	0.169	0.866	(−0.073, 0.086)
	Socioeconomic status × marital quality	−0.05	0.017	−2.878	0.004	(−0.080, −0.015)
Female	Marital quality	0.30	0.034	8.802	0.000	(0.235, 0.370)
	Socioeconomic status	−0.11	0.042	−2.509	0.012	(−0.188, −0.023)
	Socioeconomic status × marital quality	−0.01	0.015	−0.473	0.636	(−0.038, 0.023)

**Figure 3:**
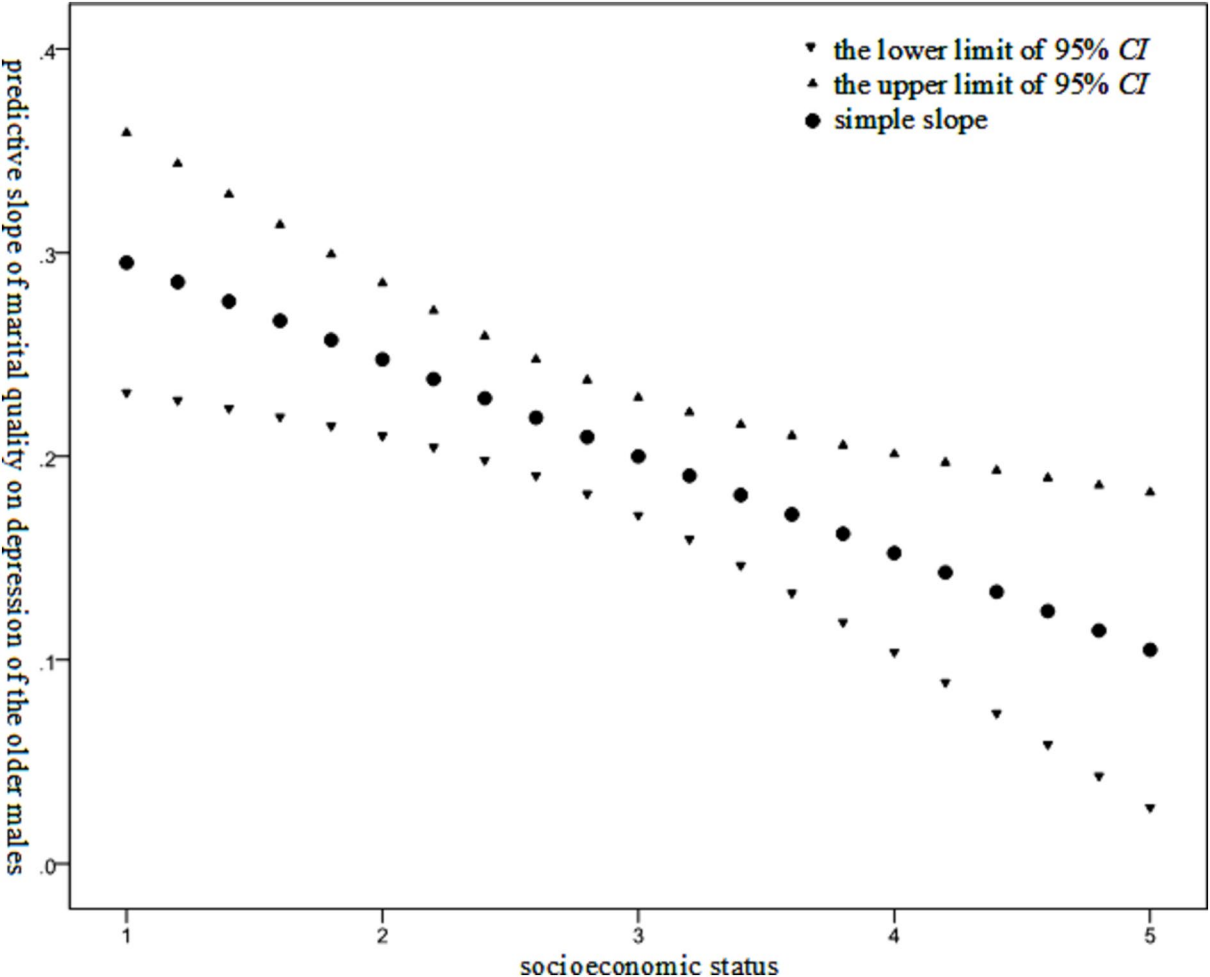
Moderating effect of socioeconomic status on the relationship between marital quality and depression level among the older males.

As presented in Table 3, it was indicated that marital quality exerted a statistically significant influence on the depression level among the older adults of different genders. Specifically, a poorer marital quality was associated with a higher level of depression among the older adults. The interaction effect between marital quality and socioeconomic status on the depression level was statistically significant only within the older males. The Johnson-Neyman technique was employed to examine the moderating effect of the socioeconomic status of the older males. The results are illustrated in Figure 3.

As illustrated in Figure 3, the effect of marital quality on the depression level of the older males was found to diminish as the socioeconomic status of the older males increased.

## 4 Discussion

In the present study, longitudinal data were utilized to perform a cross-lagged analysis of the relationship between marital quality and depression level among the older adults. The results demonstrated that the autoregressive coefficients for both paths were statistically significant, suggesting the existence of a time cumulative risk effect on both depression level and marital quality among the older adults, thereby necessitating appropriate intervention. Furthermore, an interactive influence was observed between depression level and marital quality among the older adults, with each variable exhibiting a causal relationship. On the one hand, a higher level of depression can diminish the marital quality and social functioning of the older adults, as well as undermine their emotional control and regulation capabilities. Depression predisposes the older adults to adopt negative problem-solving strategies during marital conflicts, such as crying, aggressive speech, and impulsivity. Evidently, these negative behaviors can disrupt the normal communication between spouses and subsequently degrade marital quality (Shi et al., 2014). On the other hand, marital quality can have an impact on the depression level of the older adults. Marital disharmony can readily place the couple in a state of acute and chronic stress over an extended period, culminating in psychological exhaustion (Zainab et al., 2012). Conversely, through spousal supervision and support, a healthy marriage can convey positive information to the individual, foster a healthy lifestyle, and more effectively integrate the individual’s social resources, thereby being more conducive to mental well-being (Bjereld et al., 2015; Yara et al., 2018).

In the current study, it was determined that the socioeconomic status of the older females moderated the influence of depression level on marital quality. Specifically, as the socioeconomic status of older females increases, the negative impact of depression level on marital quality becomes more pronounced. Generally, a higher socioeconomic status affords the older females greater influence in family decision-making and a stronger sense of power equity within the family. In the context of traditional Chinese culture, most of the older males adhere to the concept of “male-centeredness,” and a wife’s elevated authority might lead to a reduction in the husband’s marital tolerance. In such situations, when the older females experience symptoms such as low mood, hypersensitivity, suspicion, and internal distress due to depression, marital conflicts may intensify (Shi et al., 2014), consequently resulting in a corresponding decline in marital quality. Conversely, socioeconomic status did not exhibit a moderating effect on the impact of depression level among the older males on marital quality. Since the establishment of the People’s Republic of China, despite the government’s significant efforts to advocate gender equality and enhance women’s social status, the influence of traditional gender culture remains prevalent, which is prominently manifested in mate selection criteria. Typically, females tend to select males with a higher socioeconomic status as partners, whereas males are generally less inclined to accept females with a higher socioeconomic status than themselves (Tong and Zhang, 2015). Hence, in the majority of Chinese families, males possess a higher socioeconomic status than females. This disparity might account for the lack of a moderating role of male socioeconomic status in the relationship between depression level and marital quality.

The socioeconomic status of the older males can moderate the impact of marital quality on depression level. Specifically, as the socioeconomic status of the older males increases, the impact of marital quality on depression level decreases. Influenced by the traditional gender role culture of “men work outside, women work inside,” the self-worth and mood of males are closely associated with their socioeconomic status (Wu et al., 2022; Hu and Lei, 2023). The depression level resulting from a low-quality marriage can be alleviated by career success (Wei and Chen, 2017; Liu et al., 2020). The older males with low socioeconomic status possess a weak sense of self-worth and exhibit a stronger dependence on their spouses within the family (Jiang and Zhao, 2022). When problems arise in marital quality, the cooperation between spouses will be undermined, which will undoubtedly heighten the psychological stress of the older males, consequently leading them into a depressive state. In contrast, the impact of marital quality on depression among females is not influenced by their own socioeconomic status. Females typically place greater emphasis on family relationships. Even if they have a successful career and a high socioeconomic status, most females are reluctant to be labeled as “strong women” and continue to fulfill the role of serving both their husband and children in family life (Wang, 2019; Yang and Zheng, 2024). Hence, socioeconomic status fails to moderate the impact of marital quality on depression level among females.

The findings of this study augment and extend the insights gleaned from previous cross-sectional investigations into the relationship between marital quality and depression (Thomeer et al., 2013; Brock and Lawrence, 2014; Zhai et al., 2024). The outcomes of the cross-lagged analysis imply that timely intervention is warranted for both suboptimal marital quality and elevated depression levels to preclude the progressive deterioration of these two variables and the emergence of a self-perpetuating vicious cycle. Furthermore, for the older males ensnared in marital strife and possessing a low socioeconomic status, their families and the community should proactively engage in intervention strategies to avert the onset of depression precipitated by ailing marital quality. In the case of the older females grappling with depression and a low socioeconomic standing, their families and the community ought to dispense prompt and scientifically-grounded psychological support to forestall the further decline in marital quality induced by the deleterious effects of depression.

## 5 Conclusion

The present study, through comprehensive analysis, has arrived at the following conclusions:

1. A reciprocal causal nexus exists between marital quality and depression among the older adults.
2. Regarding the impact of depression on marital quality, the socioeconomic status of the older females functions as a moderator, in contrast to that of the older males, which fails to exhibit such a moderating effect.
3. In the context of the influence of marital quality on depression, the socioeconomic status of the older males assumes a moderating role, whereas that of the older females does not possess this moderating capacity.

Consequently, it is incumbent upon communities and families to actively attend to the older adults who exhibit low marital quality or are afflicted with depression. By disrupting the cyclical relationship between these two variables, the overall quality of life for the older adults can be enhanced.

## Data Availability

The datasets presented in this study can be found in online repositories. The names of the repository/repositories and accession number(s) can be found at: https://charls.charlsdata.com/pages/data/111/zh-cn.html.
